# Role of VDAC1 in hepatocyte apoptosis during acute liver injury in rats induced by obstructive jaundice

**DOI:** 10.22038/ijbms.2024.78454.16962

**Published:** 2025

**Authors:** Jinshan Liu, Jinlong Hu, Hongyu Xu, Liang Yan, Jiaming Yao, Baoqiang Cao

**Affiliations:** 1 Anhui No. 2 Provincial People’s Hospital Clinical College of Anhui Medical University, Hefei 230041, Anhui, China; 2 The Fifth Clinical Medical College of Anhui Medical University, Hefei 230041, Anhui, China; 3 Anhui No. 2 Provincial People’s Hospital, Hefei 230041, Anhui, China; 4 Anhui University of Science & Technology, Huainan 232001, Anhui, China; # These authors contributed equally to this work

**Keywords:** Acute liver injury, Apoptosis, Mitochondria, Obstructive jaundice, VDAC1

## Abstract

**Objective(s):**

Exploring the role of VDAC1 in hepatocyte apoptosis during acute liver injury induced by obstructive jaundice.

**Materials and Methods:**

Animal and cell models were established to investigate possible mechanisms during acute liver injury induced by OJ. Blood was collected for liver function assessment. H&E and TEM were employed to observe pathological changes in the liver tissues. Flow cytometry was used to measure the hepatocyte apoptosis. The mitochondrial MPTP assay was employed to assess the mitochondrial function of hepatocytes. IHC, western blot, and qRT-PCR were employed to determine the expression levels of VDAC1. Then, VDAC-siRNA was used to establish a knockdown model. Flow cytometry was used again to measure hepatocyte apoptosis following VDAC1 knockdown.

**Results:**

The serum of rats in the OJ group exhibited a significant increase in liver function. Irregular tissue structure and mitochondrial morphology were observed in the liver tissues of OJ rats. A significant increase in mitochondrial permeability in hepatocytes. The expression levels of VDAC1 were significantly increased in the liver tissue of OJ rats. They were also significantly increased in the hepatocytes, primarily within mitochondrial membranes, determined by western blot *in vivo *and *in vitro*. Significant increases in the rates of hepatocyte apoptosis, particularly early apoptosis, were observed in the OJ groups. However, there was a reverse in the rates of hepatocyte apoptosis after knockdown regulation of VDAC1 only within the cells of the OJ group.

**Conclusion:**

The up-regulation of VDAC in liver injury caused by obstructive jaundice may lead to increased early apoptosis of hepatocytes.

## Introduction

Obstructive jaundice (OJ), resulting from the obstruction or stenosis of the intrahepatic or extrahepatic bile duct, can initiate a series of systemic damage (1). The liver is the initial organ affected in the physiological process of OJ. The primary pathological changes in the liver of a patient with obstructive jaundice comprise structural abnormalities in the liver lobule, partial lipid degeneration of hepatocytes, and disordered arrangement of hepatocytes (2). Acute liver injury can occur in the early stages of the disease. The acute injury can become irreversible if obstruction is not promptly relieved (3). How to reverse liver damage may be a noteworthy issue. Existing studies have shown that liver damage in OJ is closely connected with hepatocyte apoptosis (4). The molecular mechanism is related to multiple factors, such as reactive oxygen species (ROS) (5), cytokine (6), intestinal endotoxemia (7), and carbon monoxide (8). There were also some studies suggesting that mitochondrial dysfunction (9) and abnormal energy metabolism (10) may be closely related to hepatocyte apoptosis during the course of OJ because of the stimulation of liver ischemia and hypoxia, which was considered to be the reason for changes in mitochondrial structure and function. The intracellular substances in the mitochondrial membrane, such as cytochrome C, may activate a series of enzymatic cascade reactions leading to cell apoptosis (11). At present, the mechanism of hepatocyte apoptosis induced by obstructive jaundice-related liver injury remains incompletely understood. More importantly, there have been no reports on whether mitochondria-related alterations are implicated in this process. Therefore, we intend to investigate this matter from the standpoint of mitochondrial functions and their associated proteins.

Mitochondria membrane permeability transition pore (MPTP) is a non-specific channel characterized by high electrical conductivity that relevant stimulating factors, such as ROS and Ca^2+^, can activate. During MPTP activation, apoptotic proteins, such as cytochrome C and Bcl-2, may be released from the mitochondria, thereby initiating cellular apoptosis. Mitochondrial MPTP was regulated by complexes formed by its membrane channel proteins. The voltage-dependent anion channel (VDAC) is a critical protein family considered an essential constituent within one of the most pivotal complexes. This complex is responsible for transporting materials within the outer mitochondrial membrane. Therefore, it may be crucial in regulating cellular survival, including cell apoptosis (12). VDAC1 is the most abundant subtype in the VDAC family, which can interact with various intracellular proteins, such as BCL-2, to affect the permeability of the outer membrane of mitochondria (13). Then, cell apoptosis occurs following the release of apoptosis-related factors from the mitochondrial intermediate space into the cytoplasm. Existing studies have shown that VDAC1 can participate in the formation of IP3Rs-Grp75-VDAC-MCU complex, thus acting as the protein group with calcium ion transport function (14). They regulate the release of calcium ions from the endoplasmic reticulum to the mitochondria, leading to ROS accumulation and MPTP opening (15), ultimately resulting in cell death (16, 17). The precise role and underlying mechanisms of VDAC1 in hepatocyte apoptosis triggered by biliary obstruction-induced liver injury remain elusive. There is also uncertainty regarding the potential for timely intervention to enhance cell survival through modulation of VDAC1. Therefore, we established animal and cell culture models to comprehensively analyze the levels of hepatocyte apoptosis and VDAC1 expression to investigate the role of VDAC1 in hepatocyte apoptosis during acute liver injury induced by OJ.

## Materials and Methods

***Cell culture and transfection***

BRL-3A rat hepatocytes were purchased from Wuhan Procell Life Technology Co., Ltd. Cells were cultured in Modified Eagle’s medium (MEM) (Gibco, USA) supplemented with 10% fetal bovine serum (FBS) (Gibco, USA) and 1% penicillin-streptomycin solution (Beyotime, China) at 37 °C in a humidified atmosphere containing 5% CO2. The culture medium was replaced every two days. BRL-3A cells were divided into two groups: Ctrl and OJ. In group OJ, BRL-3A cells were cultured in a MEM medium containing 10% serum from the OJ rats with 1% penicillin-streptomycin solution. In group Ctrl, BRL-3A cells were cultured conventionally. The cells were cultured for 24 hr and then collected for western blot, QRT-qPCR, transfection, and flow cytometry. 

Sequence-specific siRNAs were used to knock down VDAC1 transiently. We purchased pre-designed siRNAs for VDAC1 (siRNA 1, siRNA 2, and siRNA 3) and negative control siRNA (Shanghai GeneBio Co., Ltd, China). Some BRL-3A cells were transiently transfected with a transfection complex consisting of 50 nM siRNA (final concentration) and Lipofectamine™ 3000 transfection reagent (ThermoFisher, USA). They were cultured in serum-free DMEM during the transfection reaction, which lasted 6 hours. After removing the transfection complex, the cells were re-cultured in fresh MEM containing 10% FBS. The cells were allowed to grow for 48 hr and used for western blot and QRT-qPCR to detect knockdown efficiency. The VDAC1 knocked-down cells were divided into two groups: Ctrl and OJ. In group OJ, the cells were cultured in MEM medium containing 10% serum from the obstructive jaundice rats with 1% penicillin-streptomycin solution. In group Ctrl, the cells were cultured conventionally. They were cultured for 24 hr and then collected for flow cytometry. Other BRL-3A cells that play a role as negative control were transfected with negative control siRNA. They were used to repeat the above process.

***Animals and surgery***

Firstly, 30 SPF male SD rats (6–8 weeks old and 200–250 g) were acquired from Skbes Biotechnology Co., Ltd. Certificate No. SCXK (Henan) 2020-0005, Henan, China), housed at a constant temperature (25±2 °C) and allowed to feed and drink freely. All rats were randomly divided into two groups: the sham group and the OJ group. The sham group rats were only exposed to the common bile duct without ligation. The OJ group rats underwent surgery with double ligation on the confluence of the common bile duct and duodenum using 4-0 silk after sufficiently exposing their common bile duct (18). All rats were fasted and dehydrated 12 hr before surgery. After inhalation anesthesia with 3% isoflurane (Ante Animal Husbandry Technology Co., Ltd, Shandong, China), the rats were accepted surgery and resuscitated with a warm light for 30min. All rats were sacrificed seven days after surgery. Their blood and liver tissues were collected and stored at -80 ℃ for subsequent tests. This research has been approved by our institution’s Animal Care and Use Committee and conducted following the institution’s guidelines.

***Liver function test***

The rat’s blood was collected respectively and centrifuged at 300 rpm for 10 min at 20 °C, and the supernatants were collected. ALT, AST, TBIL, and DBIL levels were then measured using an automatic clinical analyzer (Chemray 800, China). 

***Isolation of rat hepatocytes***

Liver tissues were minced and resuspended in PBS. Hepatocytes were isolated by two-step liver perfusion with pronase (Worthington, USA) and Collagenase type 2 (Worthington, USA). The cells were collected into centrifuge tubes using a 200-mesh screen. Afterward, cells were separated using gradient centrifugation (19). The isolated hepatocytes were collected for flow cytometry.

***Flow cytometry***

The isolated rat hepatocytes and BRL-3A rat hepatocytes were digested by trypsin without EDTA (Gibco, USA) and collected as cell suspension. The suspension was centrifuged. Then, the supernatant was discarded, and the sediment was washed twice with PBS. All cells were detected using the AnnexinV-FITC/PI Apoptosis Detection Kit (Nanjing Kaiji Biology, China). Firstly, 500 ul Binding Buffer was added to the sediment to resuspend the hepatocytes. Then, 5 ul AnnexinV-FITC and 5 µl PI were added to the supernatant sequentially and mixed sufficiently with the hepatocytes to react at 25 ℃ for 15 min in a dark room. Finally, the apoptosis rate of hepatocytes was detected by a Flow cytometer (Agilent, USA).

***Transmission electron microscope (TEM)***

According to the literature (20), the rat’s liver tissues were divided into multiple small pieces with a size of less than 1.0 × 1.0 × 1.0 mm within 60 s after leaving the rat’s body. The pieces were fixed in 3% glutaraldehyde solution (Servicebio, China) at 4 °C for 2 hr and washed four times with phosphate buffer (Servicebio, China) for 1 hr each time. The fixed pieces of liver tissues were stained with 1% osmium acid solution (Servicebio, China) for 2 hr and rinsed three times with ddH_2_O for 10 min each time. The stained pieces were stained again with 2% uranyl acetate aqueous solution (Servicebio, China) for 2 hr. Then, they were dehydrated gradiently twice with 50%, 70%, 80% and 90% acetone solution (Servicebio, China) respectively for 10 min each time and dehydrated repeatedly with 100% acetone (Servicebio, China) twice for 15 min each time. The dehydrated pieces were embedded with a mixture composed of equal volumes of 100% acetone and embedding agent (Servicebio, China) at 25℃ for 2 hr and embedded again with a pure embedding agent at 30 °C for the whole night. An ultramicrotome (Leica, Germany) was used to slice the embedded liver tissues into thin sections with a thickness of 70~100 nm. The sections were stained with pure uranyl acetate for 30 min and rinsed thrice with ddH_2_O. Then, they were stained again with lead citrate (Servicebio, China) for 10 min and rinsed thrice with ddH_2_O. After the sections were dried, they were observed and captured by transmission electron microscope (Thermofly, USA).

***Separation of mitochondria***

Mitochondria were separated using a tissue mitochondria Isolation Kit (Beyotime, China). A small piece of rat liver tissue weighing 1g was washed with PBS and then divided into very fine fragments in a culture dish placed on ice. 10ml mitochondrial isolation reagent A was added to the culture dish above. Then, the mixture was transferred to a pre-cooled homogenizer and homogenized ten times in an ice bath. 5 ml mitochondrial isolation reagent B was added to the bottom of a new centrifuge tube. Then, 5ml homogenate above was transferred to the reagent B and centrifuged at 600 rpm for 5 min at 4 °C. The supernatant was collected and transferred carefully to another centrifuge tube and centrifuged at 11,000 rpm for 10 min at 4 °C. The isolated mitochondria were obtained from the precipitate and used for MPTP and WB. 

***Mitochondrial permeability transition pore (MPTP) assay***

The protein concentration should be detected before taking the MPTP assay to monitor the isolated mitochondrial quality. The mitochondrial samples were transferred into a 96-well plate with 20 µl per well. Their protein concentration was determined using a BCA kit (Beyotime, China). The MPTP detection kit (Shanghai Jiemei Gene, China) was used for the following assay. Firstly, 170 µl pre-warmed GENMED buffer (reagent A) was added into each well containing 20 µl mitochondrial sample. They were mixed well at room temperature. A multifunctional microplate reader (Molecular Devices, USA) was used to immediately detect the initial readings (OD1) at 540 nm. Next, 10 µl of GENMED induction solution (reagent B) was added to each well after 1 min and mixed well. The readings after 10 min (OD2) were detected as above. The change of absorbance (△OD) was calculated as OD1 minus OD2. The △OD was recorded as the opening level of MPTP.

***H*** ***&*** ***E and Immunohistochemistry (IHC)***

Liver tissues were fixed in a 4% formaldehyde solution, embedded in paraffin, and sliced into 5-micron sections. To observe the histopathologic changes, hematoxylin and eosin staining were performed. Histological features were observed and captured by an optical microscope. 

The rat liver tissue sections were obtained in the same way as above. They were repaired with 0.01M citrate buffer solution (Servicebio, China). The activity of their endoperoxidase was blocked by 3% H_2_O_2_. Then, the sections were sealed and reacted with anti-VDAC1 antibody (1:100) (Abcom, China) overnight at 4 °C. HRP-labeled goat anti-mouse secondary antibody (1:200, Servicebio, China) was added to the sections and incubated with them for 30 min at 37 °C. Freshly, a DAB solution (Servicebio, China) was used to complete the color reaction. When the positive signal could be clearly observed, the sections were restained, dehydrated, and sealed. Finally, they were observed under the optical microscope.

***Western blot***

Liver tissues, BRL-3A cells, and isolated mitochondria were collected and lysed by RIPA buffer (Servicebio, China). Membrane-cytosol protein was extracted by a Membrane and Cytosol Protein Extraction Kit (Beyotime, China). Mitochondrial protein was extracted by a Mitochondrial Protein Extraction Kit (KEYGEN, China). Then, the protein concentration was established using a BCA kit (Beyotime, China). Protein samples were separated by electrophoresis on a 10% SDS-PAGE gel (Servicebio, China) and transferred to a PVDF membrane (Servicebio, China). Blotted membranes were placed in a blocking solution of 5% non-fat milk for 50 mins and then incubated overnight at 4 °C with the anti-VDAC1 antibody. Then, the membranes were washed thrice with TBST for 10 min each and incubated with the appropriate secondary antibodies. Lastly immunocomplexes were visualized using a Tanon™ Femto Sig ECL Chemiluminescent Substrate (Tanon, China) with exposure of the transfer membrane to X-ray film. The following antibodies were used: anti-VDAC1 (1:1000) (Proteintech, China), anti-β-Actin (1:1000) (Proteintech, China), anti-COX-IV (1:1000) (Proteintech, China), and anti-ATP1A1 (1:1000) (Proteintech, China).

***QRT-qPCR***

Total RNA was extracted from cells using the Trizol reagent (Absin, China), and then it was reversely transcribed to cDNA using the Transgen Reverse Transcription Kit (TransGen Biotech, China). Then, mRNA expression was analyzed using SYBR Green I real-time PCR (relative quantification). The detection machine is lightcycler96 (Roche, Swiss). β-Actin was used as the normalization control. The results were calculated using the 2^-ΔΔCt ^method. 

The primer sequences used were as follows: VDAC1 forward, 5′-TCTGGTGCTTGGCTATGAGG-3′and reverse, 5′-AAGCTGGAATTCGTCCGTCT-3′.

β-Actin forward, 5′-TCTATCCTGGCCTCACTGTC-3′ and reverse, 5′-CAGTCCGCCTAGAAGCATTTG-3′.

***Statistical analysis***

Statistical significance analysis was performed by GraphPad Prism 5.0 software using Student’s t-test or ANOVA. Each group of experiments was repeated three times. All data were described as mean ± standard deviation, and *P*<0.05 was considered statistically significant.

## Results

***Liver function injury induced by OJ***

We examined the TBIL, DBIL, ALT, and AST levels in rats’ serums to evaluate the liver function injury induced by OJ. As shown in Table 1, the TBIL, DBIL, ALT, and AST levels in the group OJ were significantly increased compared with the group Sham. The differences in these serum levels between the two groups were statistically significant (*P*<0.05).

***Liver tissue injury induced by OJ***

**Figure 1:**
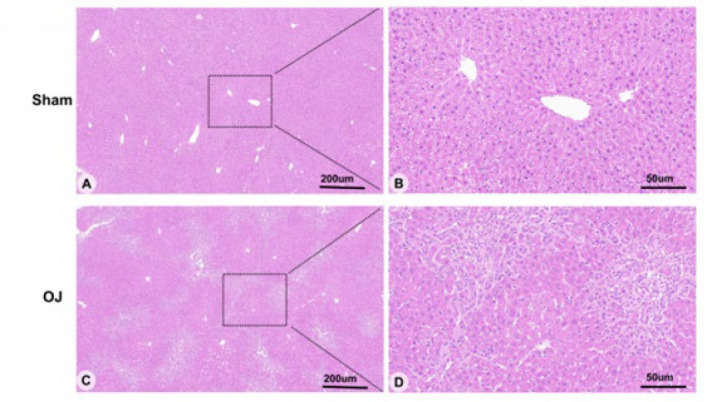
(A, B): Morphological observation of the liver of Sham group rats using H&E staining (scale bar = 200 um & 50 um). (C, D): Morphological observation of the liver of OJ group rats using H&E staining (scale bar = 200 um & 50 um)

To further observe liver tissue injury induced by OJ, we used H&E staining to detect histopathological changes in the liver tissues of rats. As shown in Figure 1 (A, B), there were no abnormal liver tissue structures in the group Sham. As shown in Figure 1 (C, D), disorder of hepatic lobules, unclear structure of hepatic cell cords, stricture of liver sinusoids, infiltration of inflammatory cells, and expansion of interlobular bile ducts in portal areas were observed clearly in the group OJ. In addition, fatty degeneration necrosis, swelling of hepatocytes, contraction of cytoplasmic, and deep staining of the nucleus were also observed clearly. These histopathological changes suggested that OJ induced liver tissue injury.

***Apoptosis of hepatocytes increased in the liver tissues induced by OJ***

**Figure 2:**
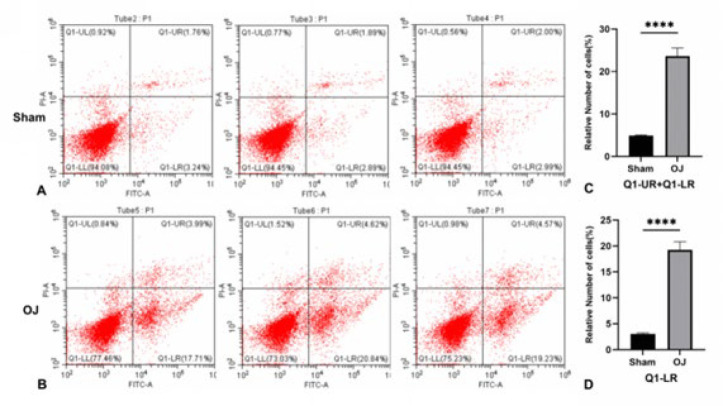
(A, B): Flow cytometry was used to detect the apoptosis results of liver cells in each group of rats, where Q1-LL represents normal living cells, Q1-UR and Q1-LR represent apoptotic cells. (C): Relative levels of apoptotic cells in the liver of two groups of rats. (D): Relative levels of early apoptotic (Q1-LR) cells in the liver of two groups of rats

To detect the type of cell death accompanied by the liver tissue injury above, we tested the apoptosis rate of hepatocytes by flow cytometry. Figure 2 (A, B) shows that the average cell survival and apoptosis rate were 94.33±0.214% and 4.92±0.124%, respectively, in group Sham. Meanwhile, these data in group OJ were 75.24±2.215% and 23.65±1.884% respectively. As shown in Figure 2 (C), compared with the group Sham, the average cell apoptosis rate was significantly increased in group OJ. As shown in Figure 2 (D), the average rate of early cell apoptosis was 3.04±0.180% in group Sham. Meanwhile, this data in group OJ was 19.26±1.565%. Compared with the group Sham, the average rate of early cell apoptosis was significantly increased in group OJ. The result suggested that apoptosis of hepatocytes, especially early apoptosis, increased in the liver tissues induced by OJ.

***Disturbed structure and enhanced MPTP permeability of mitochondria in the liver tissues induced by OJ***

**Figure 3:**
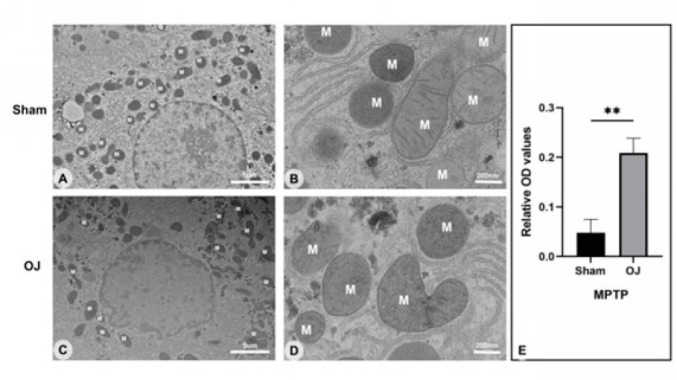
(A, B): Ultrastructural observation of the liver of Sham group rats using transmission electron microscopy (scale bar = 5 um & 200 nm). M, mitochondrion. (C, D): Ultrastructural observation of the liver of OJ group rats using transmission electron microscopy (scale bar = 5 um& 200 nm). (E): Ratio of absorbance values at wavelength 450 nm was measured biochemically for MPTP in the OJ and Sham groups using a FlexStation 3 multifunctional enzyme marker

MPTP: Membrane permeability transition pore; OJ: Obstructive jaundice

To further investigate the ultra microstructure changes accompanied by the liver tissue injury induced by OJ, we observed the ultra microstructure of rat liver tissues under TEM. These microstructure changes were mainly related to mitochondria and endoplasmic reticulum. As shown in Figure 3 (A, B), clear texture features of mitochondrial membranes and ridges were observed in the group Sham. As shown in Figure 3 (C, D), irregular morphology of mitochondria, fragmentation of them, and disappearance or unclear structure of mitochondrial ridges were observed in the group OJ. In addition, irregular shape and unclear boundary of hepatocyte nucleus, increase of smooth endoplasmic reticulum, and swelling of rough endoplasmic reticulum were also observed. These ultra microstructure changes suggested that OJ may induce the destruction of mitochondrial structure in rat hepatocytes. 

To investigate further whether mitochondrial function changes, we tested the opening level of MPTP, which is considered the main manifestation of mitochondrial function damage (21). As shown in Figure 3 (E), the OD value of the MPTP assay was increased in the group OJ (*P*<0.05). This result suggested that the OJ group’s mitochondrial membrane permeability induced by MPTP was significantly increased. OJ may induce attenuation of mitochondrial function in rat hepatocytes. 

***The expression level of VDAC1 on the mitochondrial membrane increased in Liver tissue induced by OJ***

**Figure 4:**
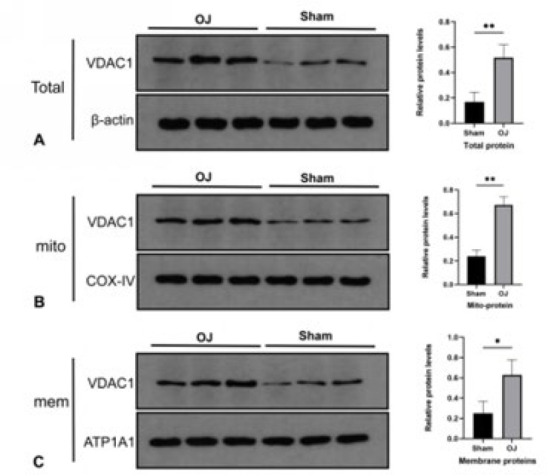
(A, B): Positive expression of VDAC1 of liver tissues in the Sham group rats was examined by immunohistochemistry (scale bar = 100 µm & 50 µm). (C, D): Positive expression of VDAC1 of liver tissues in the OJ group rats was examined by immunohistochemistry (scale bar = 100 µm & 50 µm). (E): Accumulated optical density value (IOD) of the VDAC1 was calculated in each group

OJ: Obstructive jaundice; VDAC1: Voltage-dependent anion channel 1

Considering that VDAC is a major constituent protein of the MPTP complex, we detected the expression of VDAC1 in rat liver tissues by IHC. As shown in Figure 4, the overall expression of VDAC1 in the group OJ was significantly increased compared with the group Sham (*P*<0.05). In addition, we observed that overexpressed VDAC1 was mainly distributed in the cytoplasm. They were gravelly distributed and similar to the distribution characteristics of mitochondria.

**Figure 5:**
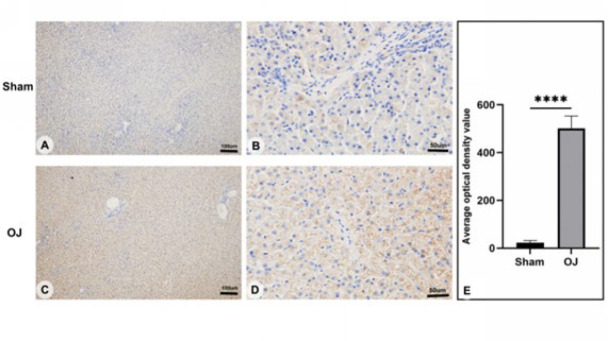
(A, B, C): Expression levels of total VDAC1, mitochondrial VDAC1, and cell membrane VDAC1 in rat livers were detected by Western blot

To investigate precisely the location of VDAC1 in the liver tissues following OJ, we detected the expression level of VDAC1 among different cellular components by western blot. As shown in Figure 5, the expression level of VDAC1 in the whole cell, on the cytoplasmic membrane, and the mitochondrial membrane were all increased in the group OJ (*P*<0.05). Although the expression level of VDAC1 on the cytoplasmic membrane was increased, the mitochondrial one increased more obviously. These results suggested that OJ may prefer to mediate hepatocyte apoptosis by regulating the level of mitochondrial VDAC1.

***Apoptosis of BRL-3A rat hepatocytes increased during co-cultured with serum from the OJ rats***

**Figure 6:**
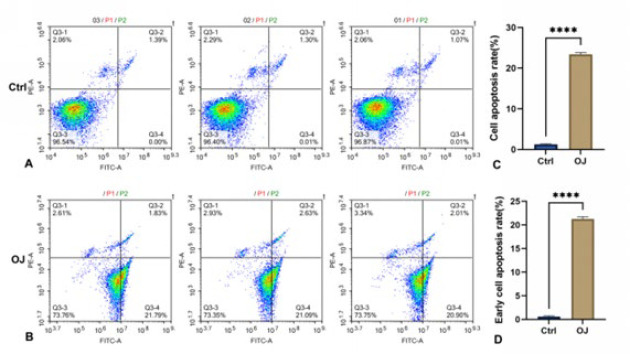
(A, B): Apoptosis of BRL-3A cells cultured in obstructive serum was detected by flow cytometry. (C): Apoptosis of BRL-3A cells cultured in obstructive serum. (D): Relative levels of early apoptotic cells in the BRL-3A cells

To eliminate the interference influence of non-hepatocytes in liver tissue, it is necessary to establish a model based on a rat hepatocyte line to simulate the process of OJ *in vitro*. We successfully established the model using a method of co-culture BRL-3A rat hepatocytes with serum from the OJ rats. We continued to test the rate of apoptosis of hepatocytes by flow cytometry. As shown in Figure 6 (A, B), the average cell survival and apoptosis rates were 73.62±0.234% and 23.42±0.442% in group OJ, based on the above model. Meanwhile, these data were 96.60±0.241% and 1.26±0.161% in group Ctrl, in which the BRL-3A rat hepatocytes were cultured without OJ rats’ serum. As shown in Figure 6 (C), compared with the group Ctrl, the average cell apoptosis rate was significantly increased in group OJ (*P*<0.05). As shown in Figure 6 (D), the average rate of early cell apoptosis was 0.01±0.005% in group Ctrl. Meanwhile, this data in group OJ was 21.26±0.469%. Compared with the group Ctrl, the average rate of early cell apoptosis was significantly increased in group OJ. The result suggested that apoptosis, especially early apoptosis, increased in the model of OJ *in vitro*.

***The expression level of VDAC1 on mitochondrial membrane increased in the model of OJ in vitro***

**Figure 7:**
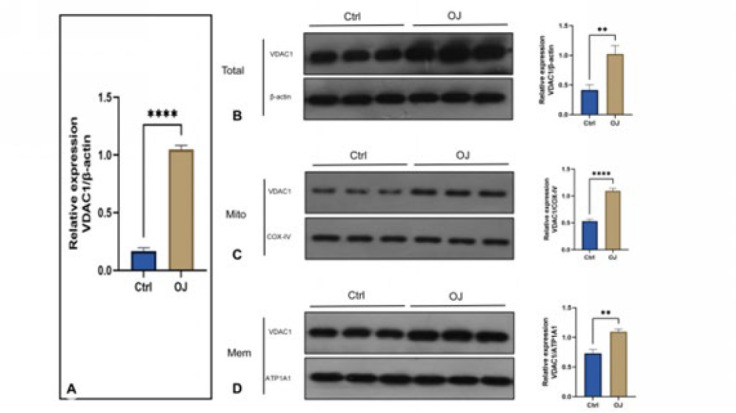
(A): Expression level of BRL-3A cells cultured in obstructive serum was detected by QPCR. (B, C, D): Expression levels of total VDAC1, mitochondrial VDAC1, and cell membrane VDAC1 in BRL-3A cells cultured in obstructive serum were detected using western blot

To verify whether over-expression of VDAC1 still exists in the BRL-3A rat hepatocytes cultured with OJ rats serum, we detected the expression level of VDAC1 in the model of OJ *in vitro* by qRT-PCR and western blot. As shown in Figure 7 (A), the mRNA expression of gene *VDAC1 *increased in BRL-3A rat hepatocytes in the group OJ compared to the group Ctrl. As shown in Figure 7 (B, C, D), similar to the results of protein expressions in the liver tissues, the expression level of VDAC1 in the whole cell, on the cytoplasmic membrane, and the mitochondrial membrane were all increased in the group OJ (*P*<0.05). The mitochondrial expression level of VDAC1 increased more obviously than it on the cytoplasmic membrane. All the results of qRT-PCR and western blot assays suggested again that OJ may prefer to mediate hepatocyte apoptosis by the regulated level of mitochondrial VDAC1.

***Knockdown of VDAC1 was beneficial in reducing the apoptosis rate of hepatocytes induced by OJ***

**Figure 8:**
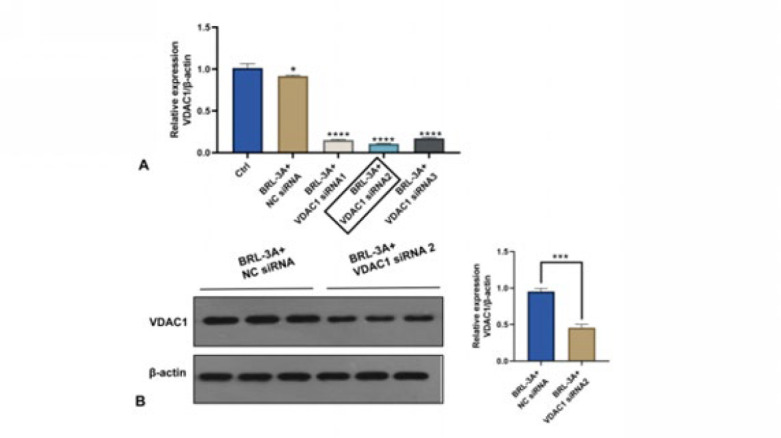
(A): The knockdown effect of three groups of siRNA on VDAC1 in BRL-3A cells was detected by QPCR. (B): The knockdown effect of the siRNA2 on VDAC1 in BRL-3A cells was detected by QPCR

To further investigate the possible role of VDAC1 in the process of hepatocyte apoptosis induced by OJ, a knockdown assay based on sequence-specific siRNAs targeting the VDAC1 gene was designed and conducted. As shown in Figure 8 (A), qRT-PCR was used to detect the knockdown efficiency of three pre-siRNAs. The results showed that the knockdown effect of BRL-3A+VDAC1 siRNA2 was the most significant among the three. As shown in Figure 8 (B), we verified the knockdown efficiency of the VDAC1 siRNA2 at the protein level by western blot again. The results showed that VDAC1 siRNA2 significantly reduced the expression levels of VDAC1 in BRL-3A hepatocytes. Therefore, the combination of BRL-3A+VDAC1 siRNA2 was used for subsequent research on whether the apoptosis rate of hepatocytes induced by OJ could be reversed following the reduction of the expression level of VDAC1. 

**Figure 9:**
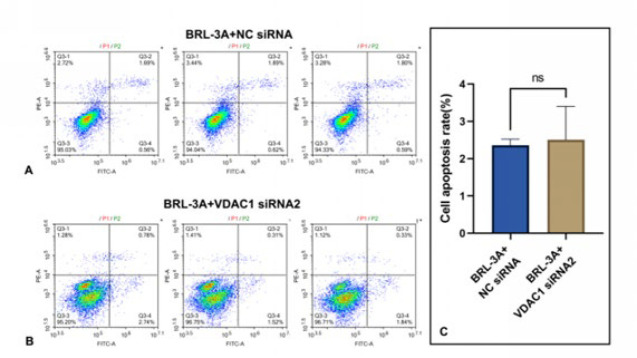
(A, B, C): In normal culture, the apoptosis rate was detected before and after VDAC1 knockdown

**Figure 10:**
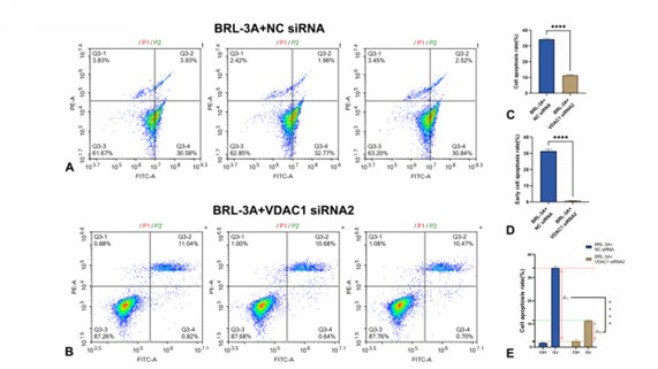
(A, B): The apoptosis rate was detected before and after VDAC1 knockdown under OJ. (C): Apoptosis rate was detected before and after VDAC1 knockdown in normal culture. (D): Early cell apoptosis rate was detected before and after VDAC1 knockdown under OJ. (E): Changes of apoptosis rate before and after VDAC1 knockdown under OJ

OJ: Obstructive jaundice; VDAC1: Voltage-dependent anion channel 1

As shown in Figure 9, in the group Ctrl, in which the BRL-3A rat hepatocytes were cultured normally, the average cell survival rate and apoptosis rate were 94.47±0.509% and 2.38±0.130%, respectively, before VDAC1 was knocked down. The data above were 96.22±0.884% and 2.51±0.780% after VDAC1 was knocked down. These results suggested that, after the VDAC1 protein was knocked down by siRNA2, the apoptosis rate did not significantly change in the hepatocytes cultured normally, meaning that the knocked-down of VDAC1 does not alter the survival status of hepatocytes under normal conditions. Under the background, As shown in Figure 10 (A, B, C), In the group OJ, in which the BRL-3A rat hepatocytes were cultured with OJ rats serum, the average cell survival rate and apoptosis rate were 63.24±0.802% and 34.20±0.736% respectively before VDAC1 was knocked down. The data above were 87.57±0.269% and 11.45±0.574% after VDAC1 was knocked down. These results suggested that, after the VDAC1 protein was knocked down by siRNA2, the apoptosis rate was significantly reduced in the hepatocytes cultured with the serum from OJ rats. Meanwhile, Figure 10 (A, B, D) shows that the average early cell apoptosis rate was 31.40±1.196% before VDAC1 was knocked down. This data was 0.72±0.092% after VDAC1 was knocked down. The early apoptosis rate was significantly reduced in the hepatocytes cultured with the serum from OJ rats after the VDAC1 protein was knocked down, meaning that the knocked-down of VDAC1 can improve the early survival status of hepatocytes under the pathological condition induced by OJ. In addition, as shown in Figure 10 (A, B, E), (average cell apoptosis rate) between group Ctrl and group OJ without knocked-down of VDAC1 was 31.82% (from 2.38% to 34.20%). While, the data after knocked-down of VDAC1 was 8.94% (from 2.51% to 11.45%). Compared with group Ctrl, even though both of the apoptosis rates of group OJ increased with or without the knocked-down of VDAC1, there were significant differences in the magnitude of the increase. The degree of increase with knocked-down was significantly lower than another one without knocked-down (8.94% VS 31.82%). These results suggested that the knocked-down of VDAC1 could more effectively reduce the degree of increase in hepatocyte apoptosis caused by OJ. Therefore, we speculated that knocked-down of VDAC1 may be an effective method to protect liver cells from harm caused by OJ. In other words, VDAC1 is expected to become an effective target for alleviating the liver injury induced by OJ.

## Discussion

Obstructive jaundice is a prevalent clinical pathological condition, primarily resulting from partial or complete obstruction of extrahepatic or intrahepatic bile ducts (22). Impaired bile flow into the intestine leads to hepatic cholestasis and subsequent elevation of bilirubin levels in the bloodstream (23). This phenomenon of bile reflux initiates a cascade of pathophysiological changes, encompassing aberrant biochemical markers, compromised immune system functionality, and impaired organ function (24, 25). Notably, impaired hepatic function becomes particularly prominent. During obstructive jaundice progression, normal metabolism is disrupted, leading to excessive generation of reactive oxygen species (26) and endotoxin lipid peroxidation (27). These events trigger a series of reactions, including inflammatory factor production (28), cytokine release (29), and altered energy metabolism due to mitochondrial dysfunction (30). Ultimately, these alterations culminate in hepatocyte ischemia, hypoxia, and acidosis development. Consequently, the liver’s structure and function are compromised (31). 

The serum levels of TBIL, DBIL, ALT, and AST in group OJ exhibited a significant increase in the present study. Additionally, the structure of the rat liver in group OJ was seriously damaged. These results indicate that OJ can seriously influence the function and structure of the liver. Schwabe RF et al. observed that acute liver injury resulting from various diseases, such as alcoholic liver disease, non-alcoholic fatty liver disease, and viral hepatitis, is characterized by extensive hepatocyte apoptosis, leading to the death of hepatic parenchymal cells (32). However, the potential association between hepatic apoptosis and acute liver function injury induced by obstructive jaundice remains uncertain. To investigate the potential correlation, we conducted experiments to evaluate the rate of hepatocyte apoptosis in both *in vitro *and* in vivo* to observe a significant increase in hepatocyte apoptosis induced by OJ. Meanwhile, we observed for the first time that these apoptotic cells were predominantly in an early stage of apoptosis. Early apoptosis is the initial stage of cell apoptosis, characterized mainly by changes in cell morphology and biological behavior. Late apoptosis is the advanced stage, characterized mainly by a pronounced process of cellular demise and the disintegration of cellular organelles. Substantial alterations in mitochondrial membrane potential are crucial in guiding cells through the transition from early to late apoptosis (33). Wang J et al. discovered that endogenous signaling pathways are sequentially activated in the event of cellular damage. Initially, the pro-apoptotic proteins Bak and Bax, which interact with Bcl-2, become active. These proteins initiate the degradation of the cell’s energy-producing organelles, mitochondria, leading to a decrease in mitochondrial membrane potential and the release of crucial proteins. Once the outer membrane of mitochondria becomes permeable, apoptosis progresses to an irreversible stage (34). In other words, early cellular apoptosis is potentially reversible, whereas late cellular apoptosis is deemed irreversible. However, the mechanism by which cells progress from early to late apoptosis remains to be further elucidated, and we plan to investigate this issue from the perspective of mitochondria. 

The pathways that activate cell apoptosis encompass both exogenous and endogenous routes. Both of these pathways can produce tBid (shortened Bid) by cleaving precursor proteins Bid, which is subsequently translocated to the mitochondria to initiate the release of apoptotic proteins (35). The release of pro-apoptotic proteins from mitochondria represents a pivotal event in the signaling cascade leading to programmed cell death, a process tightly regulated by the Bcl-2 family of proteins (36-38). For instance, the BH3-only protein domain and Bid are cleaved by multiple proteinases to generate truncated Bid, which subsequently facilitates Bax insertion/oligomerizatiaon onto the outer mitochondrial membrane, resulting in the formation of pores in the mitochondrial membrane and the release of proteins into the intermembrane space (39,40). Consequently, mitochondria play a crucial role in the cellular apoptosis pathway (41). Due to the location of mitochondrial apoptosis proteins in the interstice between the inner and outer membranes of the mitochondria, when the permeability of the outer mitochondrial membrane increases, it can lead to the release of mitochondrial apoptosis proteins (42). Hence, we employed transmission electron microscopy to examine the mitochondria of rat liver cells and observed significant swelling and abnormal changes in morphology. The results from the MPTP assay also indicated an increase in mitochondrial permeability. These indications suggested that modifying mitochondrial permeability may play a pivotal role in the hepatic injury resulting from hepatocyte apoptosis in obstructive jaundice. However, the molecular mechanisms that lead to mitochondrial permeability transition remain not fully elucidated. We intended to detect mitochondrial-related membrane proteins to delve deeply into their molecular mechanisms.

MPTP is a non-specific channel characterized by high electrical conductivity that can be activated by relevant stimulating factors, such as reactive oxygen species and Ca^2+ ^(43). Extensive activation of MPTP results in the liberation of apoptotic proteins from mitochondria, such as cytochrome C and Bcl-2, thereby triggering cellular apoptosis (44). MTPT refers to mitochondrial membrane permeability, regulated by a complex of the related membrane channel proteins. VDAC is a critical constituent protein within one of the most pivotal complexes (45). VDAC comprises three subtypes: VDAC1, VDAC2, and VDAC3. Among these subtypes, VDAC1 exhibits significant advantages in expression. The function of VDAC1 involves regulating the channels for ion, ATP, and metabolic product transport in and out of the mitochondria, particularly interacting with proteins about cell apoptosis (46). The mitochondrial cytochrome C and other apoptosis-related proteins can translocate into the cytoplasm via the pore formed by the interaction between VDAC1 and BAX/BAK, thereby triggering cellular apoptosis (47). VDAC1 might be intricately associated with cellular apoptosis. To further investigate the potential relationship between VDAC1 and cell apoptosis in liver injury induced by obstructive jaundice, we first employed IHC to assess the expression of VDAC1 protein in the liver tissues. Then, we employed western blot to determine the expression of VDAC1 protein in both *in vitro *and* in vivo*. We observed a widespread distribution of VDAC1 in the cytoplasm, and the expression level of VDAC1 was significantly higher in the OJ group of rats than in the Sham group within the liver. This distinction was especially noticeable in the expression level of mitochondrial VDAC1 protein. These findings indicated that up-regulation of VDAC1 protein, especially mitochondrial VDAC1 protein, is involved in the process of hepatocyte apoptosis triggered by liver injury caused by OJ. Excessive activation of VDAC1 resulted in alterations in mitochondrial structure and function, ultimately leading to an augmented early apoptosis of hepatocytes. However, the molecular mechanism responsible for VDAC1-induced early apoptosis of hepatocytes remains poorly understood, and the potential for timely intervention on VDAC1 to promote cell survival is also uncertain.

However, considering the intricate nature of the hepatic tissue environment (48), our focus was directed towards the phenomenon of hepatocyte apoptosis in the context of obstructive jaundice. To minimize non-hepatocyte’s impact on liver tissue, a rat hepatocyte cell line model was required to replicate the *in vitro* OJ process. We established the model by co-culturing BRL-3A rat hepatocytes with OJ rat serum. Subsequently, we knocked down VDAC1 by a specific sequence of siRNA to observe the relationship between VDAC1 and liver cell apoptosis. The experimental findings indicated that the down-regulation of VDAC1 protein expression did not significantly alter the apoptosis rate in normal cultured hepatocytes, suggesting that it had no impact on the cellular survival status in the absence of bilirubin. However, the expression of VDAC1 in the OJ model is currently under investigation to determine its potential association with cellular apoptosis. Following the down-regulation of VDAC1 protein expression, the apoptosis rate in liver cells cultured with OJ rat serum significantly decreased. The decline in apoptosis rate was primarily observed in early apoptosis. These findings suggested that the down-regulation of VDAC1 can effectively attenuate hepatocyte apoptosis induced by OJ. 

This study conducted a series of experiments on the liver and serum of rats following biliary ligation, including flow cytometry, TEM, MPTP, IHC, and western blotting. A cell model of obstructive jaundice was established to investigate the direct correlation between VDAC1 and hepatocyte apoptosis. Based on a series of findings above, we found that the biliary obstruction resulting from common bile duct ligation may be attributed to the modulation of VDAC1 protein in mitochondria, leading to structural and functional impairment of mitochondria and ultimately culminating in hepatocyte apoptosis, particularly early apoptosis. Stimulatingly, early-stage cell apoptosis can be reversed by modulating the expression of VDAC1. Therefore, we hypothesized that down-regulation of VDAC1 could serve as an efficacious strategy for safeguarding hepatic cells against the detrimental effects of OJ. In conclusion, we have observed for the first time that the up-regulation of VDAC in liver injury caused by obstructive jaundice results in an augmentation of early apoptosis of hepatocytes. VDAC1 was expected to become an effective target for alleviating the liver injury induced by OJ.

Nevertheless, this study also exhibits certain limitations. Firstly, the molecular mechanism of VDAC1 in regulating cell apoptosis remains to be thoroughly investigated in this experiment. Additionally, no other apoptotic proteins have been identified at present. Secondly, we have not conducted any human-related studies to validate this mechanism further. Finally, the challenges associated with targeting VDAC1 therapeutically remain substantial. These tasks constitute the focal points of our research group’s future endeavors.

 Surgery is considered to be the primary treatment method for obstructive jaundice (49). However, the surgery carries a higher risk (50). The surgical procedure may lead to a range of complications, including impaired wound healing, gastrointestinal bleeding, hyperglycemia, and multi-organ dysfunction, all of which can significantly impact the patient’s quality of life (51). Hence, the VDAC1 protein holds significant clinical implications for investigating the mechanism of liver cell damage induced by obstructive jaundice. It can be inferred that obstructive jaundice may cause structural and functional impairment in mitochondria and cell apoptosis through up-regulation of VDAC1 protein expression on the outer mitochondrial membrane, offering a novel avenue for clinical management of obstructive jaundice. This innovative approach carries important implications for reducing patient treatment expenses, mitigating suffering, and potentially introducing new therapeutic options to patients in the future.

**Table 1:** The ALT, AST, TBIL, and DBIL results in the serum of rats in OJ and Sham groups (X±S, n=15)

	ALT (U/l)	AST (U/l)	TBIL (U/l)	DBIL (U/l)
Sham	44.75±7.05	140.12±38.55	10.75±4.69	9.42±3.86
OJ	179.30±37.35	704.59±246.53	163.64±42.45	139.94±22.47
t	7.054	4.527	7.161	11.459
p	0.000***	0.004**	0.000***	0.000 ****

Note: Compared with Sham group, **P<0.05, ***P<0.01

ALT: Alanine aminotransferase; AST: Aspartate aminotransferase; TBIL: Total bilirubin; DBIL: Direct bilirubin

OJ: Obstructive jaundice

VDAC1: Voltage-dependent anion channel 1

VDAC1: Voltage-dependent anion channel 1

VDAC1: Voltage-dependent anion channel 1

VDAC1: Voltage-dependent anion channel 1

## Conclusion

Our research has confirmed for the first time that the up-regulation of VDAC in liver injury caused by obstructive jaundice may lead to increased early apoptosis of hepatocytes. Therefore, VDAC1 has the potential to be an effective target for alleviating liver injury caused by obstructive jaundice.

## Data Availability

All relevant data can be made available for bona fide researchers upon request from the authors.

## Declaration

We have not used any AI tools or technologies to prepare this manuscript.
